# Antiapoptotic Actions of Methyl Gallate on Neonatal Rat Cardiac Myocytes Exposed to H_**2**_O_**2**_

**DOI:** 10.1155/2014/657512

**Published:** 2014-01-12

**Authors:** Sandhya Khurana, Amanda Hollingsworth, Matthew Piche, Krishnan Venkataraman, Aseem Kumar, Gregory M. Ross, T. C. Tai

**Affiliations:** ^1^Medical Sciences Division, Northern Ontario School of Medicine, Laurentian University, Sudbury, ON, Canada P3E 2C6; ^2^Department of Biology, Laurentian University, Sudbury, ON, Canada P3E 2C6; ^3^Department of Gerontology, Huntington University, Sudbury, ON, Canada; ^4^Department of Chemistry and Biochemistry, Laurentian University, Sudbury, ON, Canada P3E 2C6; ^5^Biomolecular Sciences Program, Laurentian University, Sudbury, ON, Canada P3E 2C6

## Abstract

Reactive oxygen species trigger cardiomyocyte cell death via increased oxidative stress and have been implicated in the pathogenesis of cardiovascular diseases. The prevention of cardiomyocyte apoptosis is a putative therapeutic target in cardioprotection. Polyphenol intake has been associated with reduced incidences of cardiovascular disease and better overall health. Polyphenols like epigallocatechin gallate (EGCG) can reduce apoptosis of cardiomyocytes, resulting in better health outcomes in animal models of cardiac disorders. Here, we analyzed whether the antioxidant N-acetyl cysteine (NAC) or polyphenols EGCG, gallic acid (GA) or methyl gallate (MG) can protect cardiomyocytes from cobalt or H_2_O_2_-induced stress. We demonstrate that MG can uphold viability of neonatal rat cardiomyocytes exposed to H_2_O_2_ by diminishing intracellular ROS, maintaining mitochondrial membrane potential, augmenting endogenous glutathione, and reducing apoptosis as evidenced by impaired Annexin V/PI staining, prevention of DNA fragmentation, and cleaved caspase-9 accumulation. These findings suggest a therapeutic value for MG in cardioprotection.

## 1. Introduction

Reactive oxygen species (ROS), a product of normal cellular metabolism, are usually handled effectively by the cellular defense systems, thereby having little bearing on cellular health. Cellular redox balance is maintained by antioxidant enzymes, such as superoxide dismutase and catalase, and by signaling mechanisms to conserve a state of oxidative homeostasis. However, under situations of exaggerated stress or hypoxia, the cellular defenses may be insufficient to overcome ROS overload. Oxidative stress has been clinically shown to be relevant in the progression of cardiac diseases and heart failure [1, 2]. Excess ROS can cause a variety of cellular damage including mitochondrial dysfunction, DNA damage, and ultimately lead to apoptosis with apoptosis of cardiomyocytes being critical in tissue damage and eventually heart failure. Hence, protection of cardiomyocytes and their increased survival is a putative target for cardioprotection [3].

Increasing evidence suggests that fetal hypoxia and resultant increased ROS are factors that can program for adult diseases, a phenomenon known as developmental programming; the activation of oxidative stress pathways *in utero* can program for cardiac dysfunction in adulthood such as responses to ischemia/reperfusion, cardiac function, coronary flow, and hypertension [4–7]. Hypoxia due to intrauterine stress during fetal development affects cardiogenesis and can have adverse effects on the developing heart, affecting fetal heart morphology and function [5]. In this scenario, fetal cardiomyocytes undergo hypertrophy, likely due to increased cardiomyocyte loss resulting from increased apoptosis [8]. Antioxidants such as vitamin C administered during pregnancy to rats have proven effective in preventing oxidative stress-mediated cardiovascular dysfunction in the offspring of this animal model [9, 10]. Cardiomyocytes also undergo cell death under a variety of other physiological stimuli such as oxidative stress due to hypoxia induced during cardiac ischemia and glucose limitation amongst others [11]. Further, sustained volume and pressure overload, seen in hypertension and other cardiac disorders, ultimately lead to cardiac hypertrophy and result in cell death.

Naturally occurring polyphenols are antioxidants that are well acclaimed for their protective effects particularly in situations of oxidative stress and have been associated with inhibiting cell apoptosis, increasing viability, and being cytoprotective in nature [12]. Epidemiological studies have provided strong evidence in support of improved cardiovascular health in populations with consumption of diets rich in polyphenols [13, 14]. Studies using polyphenols have shown their beneficial effects in alleviating the effects of oxidative stress or modulating signaling pathways or both thereby reducing cardiovascular disease sequelae [15–17].

The goal of the current study was to examine the effect of polyphenols on survival of cultured neonatal rat cardiomyocytes (RCMs) under oxidative stress to evaluate mechanisms of polyphenols in cardioprotection, with an emphasis on effects on oxidative stress-related parameters. Hypoxia and oxidative stress induce apoptosis in neonatal cardiac myocytes [18–21]; further, these cells have been extensively employed as a model to study the mechanisms underlying electrophysiological heart functions and responses of myocytes to various stimuli including responses to oxidative stress and ROS, making them a suitable model for this study [22]. Previously, we have shown that the polyphenol methyl gallate (MG) is capable of protecting adrenal medulla-derived neuronal rat PC12 cells against H_2_O_2_-mediated apoptosis [23]. Cell type specificity in the mode of action of polyphenols has been reported. For example, gallic acid (GA) behaves as an antioxidant and protects HeLa cells from H_2_O_2_-induced cytotoxicity; however, in A549 cells it behaves as a prooxidant and induces apoptosis [24, 25]. Similarly, in the case of epigallocatechin gallate (EGCG), its pro-oxidant properties have been demonstrated in certain cancer cell lines; however, its antioxidant and cytoprotective capability has also been well recognized [26–28]. Hence, to assess the action of polyphenols and ascertain cell-specific differences in the mode of protection from apoptosis between PC12 and neonatal RCMs, the polyphenols EGCG, GA, and MG were employed to analyze recovery of neonatal RCMs from oxidative stress induced by H_2_O_2_ or CoCl_2_ to compare with our previous work. This study shows that, similar to the PC12 cells, in RCMs too, MG can enhance cell viability, reduce ROS, increase mitochondrial stability, and restrict progression to apoptosis in neonatal rat cardiomyocytes stressed with H_2_O_2_.

## 2. Materials and Methods

### 2.1. Cell Culture

Neonatal rat cardiac myocytes (RCMs) isolated from 2-day-old rat heart ventricle were purchased from ScienCell Research Laboratories, Carlsbad, CA, USA. The cells were maintained in a humidified incubator at 37°C with 5% CO_2_, in cardiac myocyte medium (CMM) supplemented with 5% FBS, 1% cardiac myocyte growth supplement, and 1% penicillin/streptomycin (ScienCell Research Laboratories; Carlsbad, CA).

### 2.2. Cell Treatment

To determine LD_50_, various concentrations of H_2_O_2_ or CoCl_2_ were used in an MTT assay for 24 hours. Once LD_50_ was determined, oxidative stress was induced using the LD_50_ concentration for 24 hours, specifically H_2_O_2_ (800 *μ*M) or CoCl_2_ (900 *μ*M). The polyphenols were chosen based on a prior screening study performed in our lab identifying polyphenols that were able to reduce ROS in PC12 cells under oxidative stress [29]. Polyphenol concentrations were consistent with previous studies from our lab using PC12 cells; pretreatment with N-acetylcysteine (NAC) was a positive control [23, 29]. Epigallocatechin-3-gallate (EGCG, 100 *μ*M), gallic acid (GA, 50 *μ*M), methyl gallate (MG, 50 *μ*M), or NAC (5 mM) (Sigma-Aldrich; Oakville, ON) were added to RCMs, incubated for 30 minutes, and then stressed with H_2_O_2_ or CoCl_2_ for 24 hours. The downstream processing was assay/experiment specific. Cells were imaged using an inverted microscope (Nikon ECLIPSE TS100) and processed with Adobe Photoshop CS4.

### 2.3. Viability Assays

The MTT assay was used to determine LD_50_ concentrations of CoCl_2_ and H_2_O_2_ and in the polyphenol protection experiments to determine cell viability. Cells, cultured in a 96-well plates, were treated as above, and MTT dye was added at 1/10th volume (5 mg/mL in PBS) (Sigma-Aldrich; Oakville, ON). Cells were incubated at 37°C with 5% CO_2_ in the dark for 4 hours, medium aspirated, 50 *μ*L of DMSO added (Fisher Scientific; Whitby, ON), and incubated in the dark for 15 minutes. The OD_570_ was measured on a plate reader (BioTek, PowerWave XS; Winooski, VT, USA). Optical densities of samples were normalized to controls.

### 2.4. Intracellular ROS

Intracellular ROS was measured using a fluorogenic dye, CM-H_2_DCFDA (5-(and-6)-chloromethyl-2′7′-dichlorodihydrofluorescein diacetate, acetyl ester) (Invitrogen Canada; Burlington, ON) as per the manufacturer's instructions. Fluorescence was measured at 495 nm and emission at 525 nm via flow cytometry (FACS Canto II, BD Biosciences; San Jose, CA). The fluorescence from 10,000 events was averaged and relative fold changes determined by comparing with controls.

### 2.5. JC-10 Staining

Mitochondrial membrane potential was assessed by flow cytometry using a fluorogenic dye, JC-10 (Abcam; Cambridge, MA). Treated cells were loaded with JC-10 dye according to the manufacturer's instructions with modifications: spent medium was aspirated and complete medium added to scrape cells. JC-10 solution was added at equal volume and incubated in the dark at 37°C for 15 minutes prior to analysis. For the positive control, cells were incubated with FCCP (carbonyl cyanide 4-(trifluoromethoxy) phenylhydrazone), preceding the JC-10 solution. Monomeric (green) and J-aggregate (red) fluorescence were measured using the FL1 and FL2 channels, respectively, and analyzed following compensation for spectral overlap.

### 2.6. Annexin V and PI Staining

Apoptosis was measured by flow cytometry using CytoGLO Annexin V-FITC Apoptosis Detection Kit (IMGENEX Corporation; San Diego, CA) as per the manufacturer's instructions. Relative fold changes in the percentage of Annexin V^+^/PI^+^ and Annexin V^−^/PI^−^ were compared to controls.

### 2.7. Total Glutathione

Total glutathione was quantified enzymatically as per the manufacturer's instructions (Enzo Life Sciences, Plymouth Meeting, PA) and absorbance normalized to protein concentration quantified by BCA assay (Pierce, Thermo Scientific; Rockford, IL). Relative change was determined by comparing with controls.

### 2.8. DNA Fragmentation

DNA from treated and control cells was isolated as previously described [30], resolved on a 2% agarose gel, and imaged with Biorad Chemidoc XRS and Quantity One software.

### 2.9. Fluorescence Microscopy

RCMs were planted on acid washed coverslips and treated as above. The cells were washed with PBS, fixed with 3.7% p-formaldehyde, permeabilized with 0.2% Triton X100, and stained with anticleaved caspase-9 (rat specific Clone, Asp353, Cell signaling) for 1 hour. Alexa 488 conjugated secondary and DAPI were then applied for 30 minutes in the dark (Invitrogen, Burlington, ON, Canada). The coverslips were visualized on a confocal microscope (Zeiss TE2000). A minimum of 10 fields and 50 cells were counted, *n* = 3, and images processed using Adobe Photoshop CS4.

### 2.10. Statistical Analysis

Data are represented as mean ± SEM. with a minimum *n* = 3. Comparisons of treatment groups were made to controls by one-way ANOVA and Dunnett's *post hoc* test (GraphPad Prizm, La Jolla, CA). Values of *P* ≤ 0.05 were considered statistically significant.

## 3. Results

### 3.1. MTT Assay

**Figure 1:**
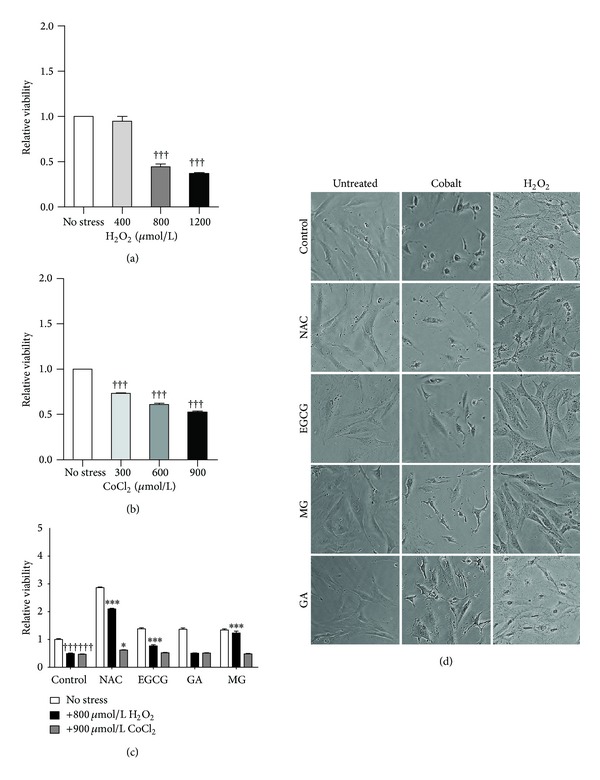
Oxidative stress affects viability and morphology of neonatal rat cardiac myocytes. LD_50_ concentrations were determined for RCMs using varying concentrations of H_2_O_2_ (a) or CoCl_2_ (b). RCMs, pretreated with 5 mM NAC, 100 *μ*M EGCG, 50 *μ*M GA, or 50 *μ*M MG, were exposed to 800 *μ*M H_2_O_2_ or 900 *μ*M CoCl_2_ for 24 hours before proceeding to MTT assay (c) or imaging (d) as described in the methods. Stressed cells versus unstressed controls (^†††^ *P* < 0.001) and versus stressed cells pretreated with NAC or polyphenols (***P < 0.001, *P < 0.05). Data is expressed as mean ± S.E.M., *n* = 3.

The MTT assay was employed to assess LD_50_ for each stressor as a function of cellular metabolic activity. Varying concentrations of H_2_O_2_ or CoCl_2_ were added to the cells and viability analyzed at 24 hours of treatment. The LD_50_ was determined to be 800 *μ*M for H_2_O_2_ and 900 *μ*M for CoCl_2_ (Figures 1(a) and 1(b), resp.). Applying the LD_50_ concentrations, rescue of viability was assessed after oxidative stress, in the presence or absence of NAC or the polyphenols EGCG, MG, or GA. NAC was used as a positive control because of its well-documented antioxidant effects, including the scavenging of ROS, stimulation of glutathione synthesis, and detoxification [31]. Viability of CoCl_2_-stressed cells did not significantly change with prior exposure to EGCG, GA, MG, or NAC (Figure 1(c)). However, pretreatment with NAC, EGCG, or MG significantly increased viability of RCMs stressed with H_2_O_2_ from 49.5% to 209.9%, 76.9%, and 123.2%, respectively (Figure 1(c)), indicating their protective effect only against H_2_O_2_ but not cobalt. The assessment of viability by the MTT assay was verified with trypan blue exclusion, since the former detects mitochondrial functionality, while the latter depends on the integrity of the limiting membrane; the data from both assays were consistent (data not shown). A morphological assessment of treated RCMs was done to visualize cellular damage. The images show that cells treated with NAC or EGCG, MG, or GA were virtually indistinguishable from controls (Figure 1(d)). In contrast, cells treated with H_2_O_2_ or CoCl_2_ appeared stressed and did not have the same attachment properties as controls. The H_2_O_2_-stressed cells appeared to lose membrane integrity with signs of membrane damage. Cells treated with CoCl_2_ appeared rounded and lost their attachment properties. In both stressors, pretreatments with NAC, EGCG, and MG treatment considerably reversed these morphological effects, while GA had little to no effect (Figure 1(d)).

Although EGCG improved viability and maintained cell integrity, the cardioprotective effects of MG was further examined due to its more potent ability to increase cell viability and considering our prior investigations on MG's ability to prevent apoptosis in PC12 cells. Also, subsequent experiments were restricted only to H_2_O_2_ since no rescue was seen with cobalt.

### 3.2. Intracellular Reactive Oxygen Species

**Figure 2:**
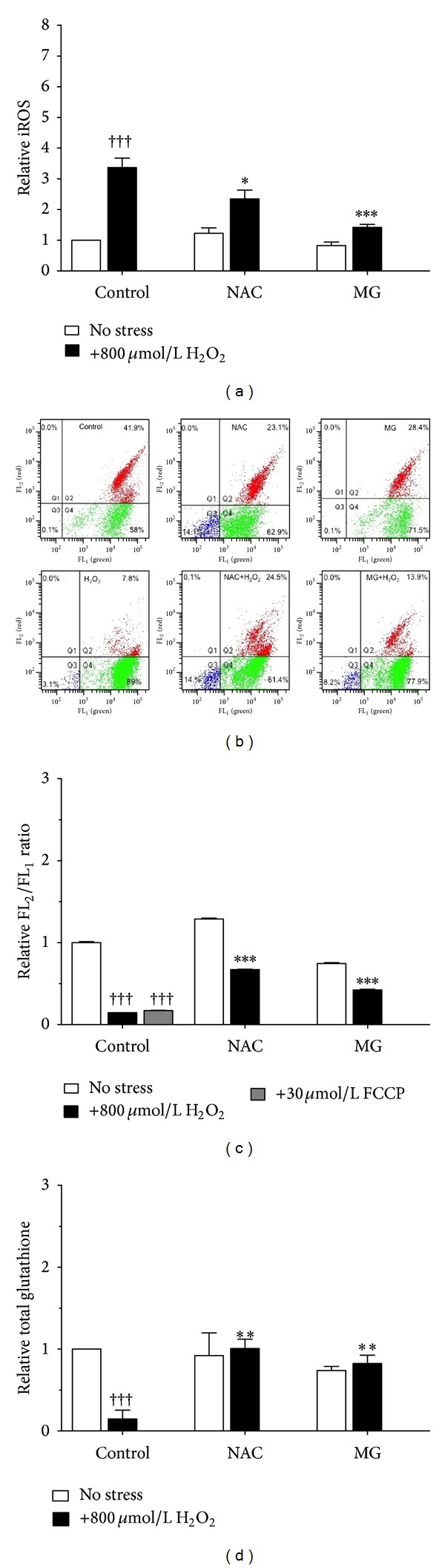
Methyl gallate represses oxidative stress. Neonatal RCMs pretreated with 5 mM NAC or 50 *μ*M MG were exposed to 800 *μ*M H_2_O_2_ for 24 hours before proceeding to measurements of either intracellular ROS (a), or mitochondrial membrane potential presented as a representative scatterplot (b) or graphically (c) and total glutathione levels as described in methods (d). Stressed cells versus unstressed controls (^†††^ *P* < 0.001) and versus stressed cells pretreated with NAC or MG (***P < 0.001, **P < 0.01, *P < 0.05). Data is expressed as mean ± S.D, *n* = 3.

Since excess H_2_O_2_ causes accumulation of intracellular ROS (iROS), which is detrimental to cellular health, the ability of MG to reduce iROS and thereby increase cell viability was examined. Flow cytometry was used to quantify iROS in treated cells compared to healthy controls. Exposure to H_2_O_2_ resulted in a 3.4-fold increase in production of iROS; both NAC and MG significantly decreased H_2_O_2_-driven generation of iROS as pretreatment with these compounds decreased iROS levels to 2.3-fold and 1.4-fold, respectively (Figure 2(a)).

### 3.3. Mitochondrial Potential

This study further evaluated whether the increased iROS in H_2_O_2_-stressed cells was accompanied by of a loss of mitochondrial potential and if MG pretreatment could affect this phenomenon. The cells were analyzed with the JC-10 dye that forms red J-aggregates in healthy cells but stays a green monomer in cells that have lost mitochondrial integrity; treatment with FCCP was used as a positive control for functionality of the JC-10 dye. The scatter plots show that majority of the cells treated with H_2_O_2_ shifted towards green fluorescence when compared to controls (Figure 2(b)). Remarkably, in cells pretreated with the antioxidants NAC or MG, a population shift to the red channel was observed indicating preservation of mitochondrial potential. Consequently, H_2_O_2_-stressed cells maintained mitochondrial potential when pretreated with either NAC or MG (Figure 2(c)).

### 3.4. Total Glutathione

The synthesis of glutathione, a key antioxidant produced endogenously by cells to overcome oxidative stress, can be modulated by polyphenols [32]. Thus, to assess the antioxidant capacity of the cells, total glutathione was measured in H_2_O_2_-stressed cells and in those that were treated with MG prior to the stressor. The level of endogenous glutathione was significantly diminished upon H_2_O_2_ exposure. However, pretreatment of stressed RCMs with NAC or MG significantly increased total glutathione to normalcy (Figure 2(d)).

### 3.5. Apoptosis Assays

**Figure 3:**
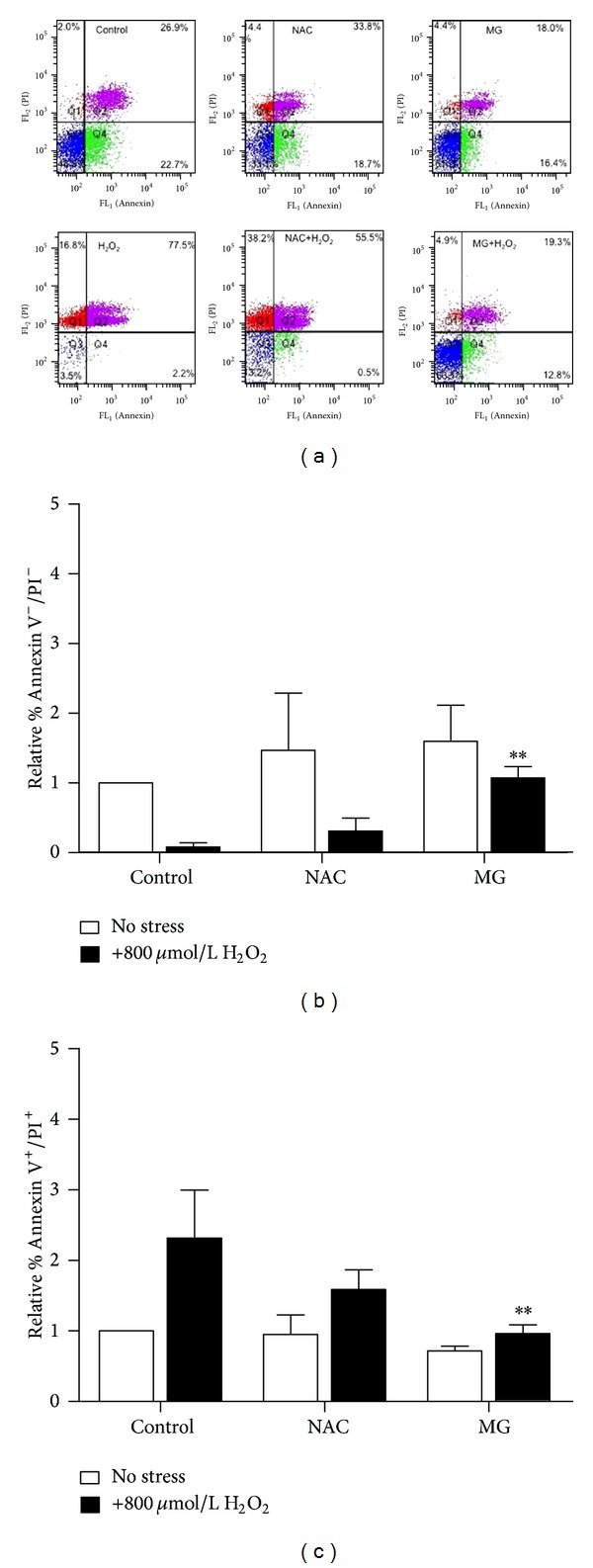
Methyl gallate protects cell viability by preventing apoptosis. Neonatal RCMs, pretreated with 5 mM NAC or 50 *μ*M MG prior to stress induction with H_2_O_2_ for 24 hours, were stained with Annexin V and PI and analyzed by flow cytometry to assess apoptotic events by scatter distribution (a). The data is graphically represented asunstained Annexin^−^/PI^−^ (healthy) RCMs (b) and late apoptotic Annexin^+^/PI^+^ cells (c). Stressed cells versus unstressed controls (^††^ *P* < 0.01) and versus stressed cells pretreated with NAC or MG (**P < 0.01, *P < 0.05). Data is expressed as means ± S.D, *n* = 3.

Numerous studies have established H_2_O_2_ as an inducer of cell apoptosis via loss of mitochondrial integrity and DNA damage. Evidence suggests that polyphenols mediate cell protective effects by interfering with apoptosis. Since MG seemed to increase viability, reduce ROS, and protect mitochondria, we wanted to further analyze whether MG could potentially affect progression to apoptosis in H_2_O_2_-stressed cells. To determine this, we used flow cytometry to quantify Annexin V^+^ and Propidium Iodide (PI^+^) populations; late apoptotic cells are the dual positive fluoresced population in Q2 while unstained cells segregate in Q3 in the scatter plots (Figure 3(a)). Majority of the control cells and MG or NAC treated cells were Annexin V^−^/PI^−^ indicating their healthy status (Figures 3(a) and 3(b)). However, H_2_O_2_-stressed RCMs showed a shift to dual stained with a 2.3-fold increase in this population when compared to the controls (Figures 3(a) and 3(c)). Eminently, MG reduced binding of Annexin V and PI in H_2_O_2_-stressed RCMs, with a significant shift from Q2 to Q3 suggesting that MG protects RCM cells from late apoptotic events (Figures 3(a) and 3(b)). NAC also demonstrated similar trends (Figures 3(a), 3(b), and 3(c)).

**Figure 4:**
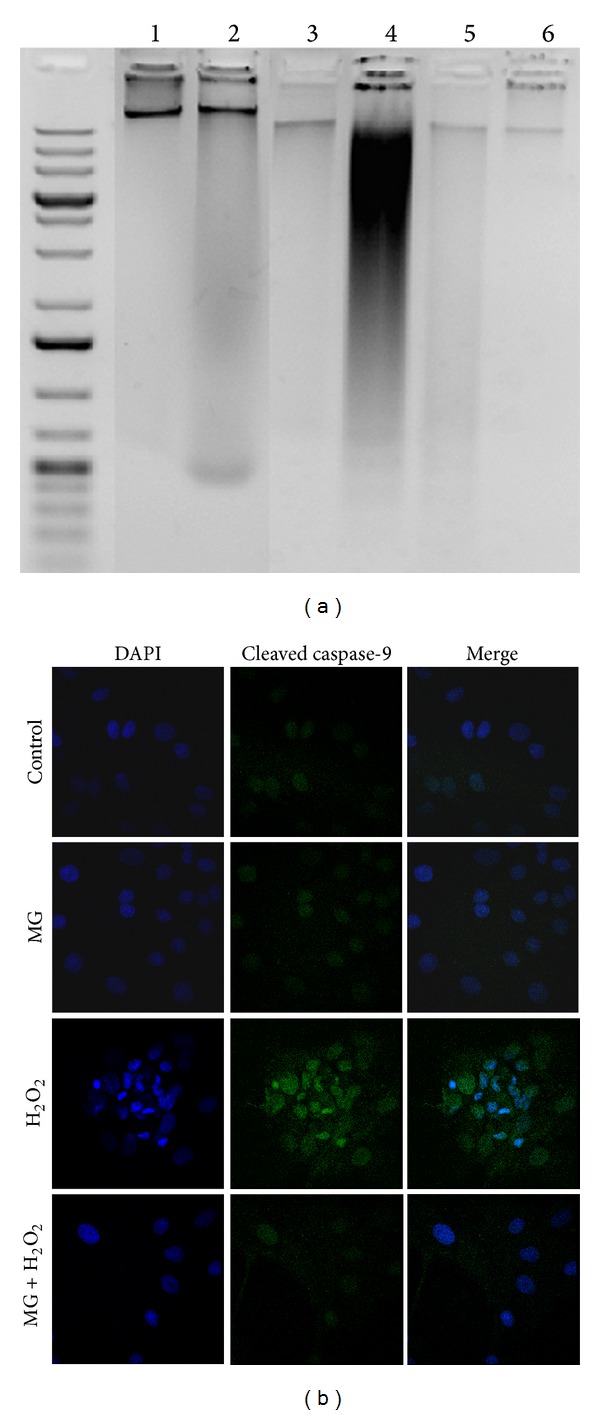
Methyl Gallate prevents DNA damage and inhibits caspase-9 activation. Total DNA was isolated from treated neonatal RCMs as described in methods. The lanes were regrouped from different parts of a representative gel (a) showing: 1Kb + ladder, Lane 1: Control, Lane 2: NAC, Lane 3: MG, Lane 4: H_2_O_2_, Lane 5: NAC + H_2_O_2_, and Lane 6: MG + H_2_O_2_. Fluorescent microscopy images of RCMs stained for cleaved caspase-9 and DAPI (b) showing: Panel 1: Control, Panel 2: MG, Panel 3: H_2_O_2_, and Panel 4: MG + H_2_O_2_.

Further DNA fragmentation assays were performed to determine whether MG is capable of protecting DNA from damage in cells undergoing H_2_O_2_-mediated apoptosis. Indeed, pretreatment of RCMs with MG protected DNA; the typical DNA laddering pattern seen in apoptotic cells was significantly rescued in cells pretreated with MG (Figure 4(a)). This corroborates the interpretation from the Annexin V/PI data that MG protects cells under oxidative stress by preventing apoptotic events. To understand a potential cellular mechanism, levels of cleaved caspase-9 were analyzed, with caspase-9 being an initiator caspase upregulated in apoptotic cells. A microscopy-based approach was used to determine the intensity of cleaved caspase-9 and nuclear architecture revealed by DAPI staining. Control cells demonstrated a weak signal in the green channel reflective of the absence of cleaved caspase-9, with healthy rounded nuclei (Figure 4(b), Control panel). The MG or NAC alone treated cells showed a similar pattern as control (Figure 4(b), MG panel and data not shown). Upon treatment with H_2_O_2_, an increase in intensity is apparent in the green channel indicative of cleaved caspase-9 accumulation, with the nucleus exhibiting a condensation of chromatin into crescent-shaped structures and increased blue intensity (Figure 4(b), H_2_O_2_ panel). Remarkably, treatment with MG rescued the cells from nuclear damage and blocked the accumulation of cleaved caspase-9 (Figure 4(b), H_2_O_2_ + MG panel); similar results were seen with NAC (data not shown). It must be noted that a 100% effect was not observed, with both healthy and unhealthy appearing cells present in the H_2_O_2_ + MG group. Therefore, cells were counted; a significant 30% reduction was calculated in the number of unhealthy cells that were observed in the MG pretreated group as compared to the H_2_O_2_ alone. Consequently, MG protects the nuclear architecture and prevents cleaved caspase-9 accumulation in H_2_O_2_-stressed cells.

Altogether, this study demonstrates the ability of MG to protect neonatal RCMs exposed to H_2_O_2_ from apoptosis by preserving mitochondrial membrane integrity, reducing ROS, increasing glutathione, protecting DNA from damage, and reducing activation of caspase-9 due to oxidative stress.

## 4. Discussion

Oxidative stress, a major contributor to cell death, has been widely implicated in cellular damage and progression to cardiovascular disease [1]. Apoptosis is a significant cause of ROS-mediated cardiomyocyte cell loss during hypoxia, prenatal stress, ischemia-reperfusion, and other cardiac disorders. Polyphenols have been widely studied for their beneficial effects in reversing the effects of ROS thereby protecting cells from death. Our data demonstrates that, of the polyphenols analyzed, none were able to restore viability of neonatal RCMs from CoCl_2_ stress. Further, only EGCG and MG restored cell viability of H_2_O_2_-stressed cells to the same as untreated controls. GA was unable to increase viability of either CoCl_2_- or H_2_O_2_-stressed cells indicating a different mechanism of action from EGCG or MG. Additionally, EGCG and MG were able to rescue only from H_2_O_2_ stress implying source-dependent specificity in protection from oxidative stress; while cobalt reacts with H_2_O_2_ via a Fenton-based reaction to increase ROS, H_2_O_2_ generates highly reactive superoxide and hydroxyl radicals via the Haber-Weiss reaction [33, 34]. Moreover, certain polyphenols like EGCG and GA can behave as prooxidants and increase intracellular ROS ([35] and data not shown). The ability of MG to reduce iROS indicates direct antioxidant potential by acting as a scavenger for ROS or an indirect mediation of ROS reduction by modulating cellular signaling pathways; both mechanisms have been reported for polyphenols [36, 37].

Glutathione (GSH) is a crucial cellular antioxidant as it interacts directly with ROS, thereby oxidizing it to GSSG, which can be eliminated or converted by glutathione reductase (GSR) back to GSH. GSH also acts as a substrate for glutathione S-transferase (GST) which complexes it with electrophilic molecules destined for removal, like end products of oxidation and other toxics. GSH is also required in the reaction for glutathione peroxidase- (GPx-) mediated detoxification of H_2_O_2_ [38]. Because of its ROS scavenging properties, as evident by overall decreased iROS in cells pretreated with MG prior to the stressor, it is plausible that increased available GSH is a direct consequence of a reduced demand to counteract ROS. The ability of MG to increase cellular GSH has also been demonstrated in MDCK cells and, by our group, in PC12 cells exposed to H_2_O_2_ stress [29, 39]. The ability of MG to increase cellular GSH content is in keeping with other polyphenols shown to increase phase II enzymes in the oxidative stress response like GSR and GPx by gene activation via antioxidant response elements (ARE); in this regard, polyphenols have been noted to have mechanisms of protection that are distinct from their antioxidant capabilities alone [32]. However, based on the current data, we cannot pinpoint the exact mechanism by which MG increases cellular GSH or mediates iROS reduction.

The MTT assay data correlates with the JC-10 assay, both indicators of mitochondrial functionality, implying that MG rescued viability by preserving mitochondrial integrity. Mitochondrial health and maintenance of mitochondrial membrane potential are critical to cell survival. The exposure of cells to H_2_O_2_ has been shown to collapse the mitochondrial membrane potential in a variety of cell types. Damage to the mitochondrial membrane is a proven central player in the initiation of intrinsic apoptotic events, with cytochrome c release from the inner mitochondrial membrane being a factor that can be modulated by ROS [40]. In the ensuing steps, caspase-9 is cleaved subsequently acting as an initiator caspase and activates effector caspases like caspase-3 [41]. Active caspase-3 ultimately leads to mobilization of caspase activated DNase (CAD) into the nucleus, DNA fragmentation, and end-stage apoptosis. Early apoptosis is marked by the exposure of phosphatidylserine groups on damaged limiting membranes, which can bind to Annexin V with high affinity. In addition, damaged membranes are permeable, thus allowing the otherwise impermeable PI to enter cells and intercalate with DNA. Healthy intact cells are Annexin V^−^/PI^−^; cells in early apoptosis are characterized by Annexin V^+^/PI^−^, while the dual stained population Annexin V^+^/PI^+^ characterizes late apoptotic/necrotic cells [42]. Our lab has previously demonstrated MG's protective abilities against apoptosis in PC12 cells, specifically in preventing activation of caspase-9 [23, 29]. In the current study, MG showed similar cytoprotection in RCMs by diminished Annexin/PI staining, reversal of DNA laddering, and reduction of caspase-9 activation.

However, not all H_2_O_2_-stressed cells showed cleaved caspase-9^+^ staining, but the endpoint of apoptosis was observed in the form of degraded DNA implying that the extrinsic pathway of apoptosis was also likely activated by H_2_O_2_. In the extrinsic pathway, the engagement of Fas ligand (Fas L) to cell surface Fas leads to Fas trimerization, followed by caspase-8 activation and recruitment of the death complex; FLIP (FLICE inhibitory protein) can inhibit cell death by the Fas/FasL pathway [43]. Previous studies showed that ROS can regulate FLICE and that H_2_O_2_ exposure can sensitize cardiomyocytes to Fas-mediated cell death [44]. However, in the current study, the role of extrinsic apoptotic factors was not examined, with the primary focus on mitochondrion-mediated intrinsic pathway. Nevertheless, polyphenols such as EGCG can modify Fas receptor expression and modulate STAT-1 phosphorylation thereby reducing apoptosis of cardiomyocytes [45]. Therefore, it is plausible that MG may have played some role in inhibiting the extrinsic pathway, demonstrated in the complete protection from DNA degradation upon MG pretreatment.

In summary, our data emphasizes the role of MG in repressing apoptosis progression in neonatal cardiomyocytes subjected to H_2_O_2_ stress by scavenging of ROS, increased endogenous glutathione, safeguarding mitochondria and cellular DNA, and inhibiting the intrinsic apoptotic pathway. In conclusion MG has the potential to combat oxidative stress in neonatal cardiomyocytes and could have therapeutic value in cardioprotection.
